# From known to the unknown: investigating an unusual outbreak of viral exanthema in a secondary school in Abeokuta, Nigeria, 2015

**DOI:** 10.11604/pamj.supp.2018.30.1.15264

**Published:** 2018-05-16

**Authors:** Magbagbeola David Dairo, Oluwaseun Ebenezer Oladeinde, Akinyode Oluyomi Bamiselu, Patrick Nguku, Joseph Asamoah Frimpong, Meeyoung Mattie Park

**Affiliations:** 1 Nigeria Field Epidemiology and Laboratory Training Programme, Nigeria; 2 Department of Epidemiology and Medical Statistics, College of Medicine, University of Ibadan, Nigeria; 3 Ministry of Health Ogun State, Nigeria; 4 African Field Epidemiology Network, Accra, Ghana; 5 Rollins School of Public Health, Emory University, Atlanta, USA

**Keywords:** Outbreak investigation, viral, exanthema, Nigeria

## Abstract

Investigating an outbreak of disease requires mastery of a set of skills and collaboration among different cadres of health workers. Although you want to focus on a specific disease, you need to keep your mind open to possibilities. This case study is based on investigation of an outbreak of rashes suspected to be measles but which proved to be otherwise. It reinforces the knowledge of the steps in outbreak investigation which should have been covered in classroom lecture or background reading. This case study is best suited for basic level of training in field epidemiology and can be completed within 2-3 hours.

## How to use this Study

**General instructions:** this case study is to be used in a classroom setting for 12-20 participants.

**Audience:** residents at the basic/frontline level in Field Epidemiology and Laboratory Training Programmes, trainees in the epidemiology course in the general public health training programmes, medical officers of health of the local government area and state surveillance officers and other health workers involved in outbreak investigations.

**Prerequisites:** participants should have prior lectures in outbreak investigation. Participants should also have basic knowledge of rates, ratio and frequencies.

**Materials needed:** laptops with Microsoft Excel (or graph paper, and pencil), white board or flip charts with markers.

**Level of training and associated public health activity:** Novice – outbreak investigation

**Time required:** 2-3 hours

**Language:** English

## Competing interest

The authors declare no competing interest.
